# Safety profile and signal detection of phosphodiesterase type 5 inhibitors for erectile dysfunction: a Food and Drug Administration Adverse Event Reporting System analysis

**DOI:** 10.1093/sexmed/qfad059

**Published:** 2023-11-29

**Authors:** Young Eun Shin, Sirikan Rojanasarot, Ana L Hincapie, Jeff Jianfei Guo

**Affiliations:** Division of Pharmacy Practice and Administrative Sciences, James L. Winkle College of Pharmacy, University of Cincinnati, Cincinnati, OH 45229, United States; Health Economics and Market Access, Boston Scientific, Marlborough, MA 01752, United States; Health Economics and Market Access, Boston Scientific, Marlborough, MA 01752, United States; Division of Pharmacy Practice and Administrative Sciences, James L. Winkle College of Pharmacy, University of Cincinnati, Cincinnati, OH 45229, United States; Division of Pharmacy Practice and Administrative Sciences, James L. Winkle College of Pharmacy, University of Cincinnati, Cincinnati, OH 45229, United States

**Keywords:** erectile dysfunction, PDE5 inhibitors, adverse events, FAERS, signal detection, sildenafil, tadalafil, vardenafil, avanafil

## Abstract

**Background:**

Phosphodiesterase type 5 inhibitors (PDE5Is) are generally well tolerated but have been associated with uncommon and significant adverse events (AEs).

**Aim:**

This study aims to investigate and compare the characteristics of AEs associated with PDE5Is used for erectile dysfunction and identify any safety signals in a postmarketing surveillance database between 2010 and 2021.

**Methods:**

A descriptive analysis was conducted for all AEs reported to the Food and Drug Administration Adverse Event Reporting System for 4 PDE5Is—avanafil, sildenafil, tadalafil, and vardenafil—indicated for erectile dysfunction between January 2010 and December 2021. The frequency of the most reported AEs and outcomes were identified. A disproportionality analysis based on proportional reporting ratio (PRR) and reporting odds ratio (ROR) was conducted for the most common and clinically important AEs to identify signals to gain insights into potential differences in safety profiles.

**Outcomes:**

The outcome measures of the study are frequency of reported AEs and outcomes following AE.

**Results:**

A total of 29 236 AEs were reported for PDE5Is during the study period. The most reported AE was “drug ineffective” with 7115 reports (24.3%). Eight safety signals were detected across the 4 drugs. Key signals were sexual disorders (PRR, 3.13 [95% CI, 2.69-3.65]; ROR, 3.24 [95% CI, 2.77-3.79]) and death (PRR, 3.17 [2.5-4.01]; ROR, 3.211 [2.52-4.06]) for sildenafil, priapism (PRR, 3.63 [2.11-6.24]; ROR, 3.64 [2.12-6.26]) for tadalafil, and drug administration error (PRR, 2.54 [1.84-3.52]; ROR, 2.6 [1.86-3.63]) for vardenafil. The most reported outcomes were other serious events with 6685 events (67.2%) and hospitalization with 1939 events (19.5%).

**Clinical Implications:**

The commonly reported AEs and detected signals may guide clinicians in treatment decision making for men with erectile dysfunction.

**Strengths and Limitations:**

This is the first comprehensive report and disproportionality analysis on all types of AEs associated with PDE5Is used for erectile dysfunction in the United States. The findings should be interpreted cautiously due to limitations in the Adverse Event Reporting System, which includes self-reports, duplicate and incomplete reports, and biases in reporting and selection. Therefore, establishing a causal relationship between the reported AEs and the use of PDE5Is is uncertain, and the data may be confounded by other medications and indications.

**Conclusion:**

PDE5Is demonstrate significantly increased risks of reporting certain clinically important AEs. While these events are not common, it is imperative to continually monitor PDE5I use at the levels of primary care to national surveillance to ensure safe utilization.

## Introduction

An estimated 30 to 50 million American men have erectile dysfunction (ED),^1^^,^^2^ with >600 000 new cases of ED expected annually in the United States.^2^ Among these men with ED, approximately 10.2 million were insured and actively seeking care for their ED symptoms in 2022.^3^ Phosphodiesterase type 5 inhibitors (PDE5Is) are recommended as first-line treatment for ED.^4^ The US Food and Drug Administration (FDA) has approved 4 PDE5Is for use: sildenafil, tadalafil, vardenafil, and avanafil. While PDE5Is are generally well tolerated, known side effects occur in about 40% of patients^5-7^ including headache, indigestion, nasal stuffiness, mild visual changes, myalgia, hypotension, and dizziness. PDE5Is are also associated with uncommon but significant events, such as nonarteritic anterior ischemic optic neuropathy, hearing loss, priapism, melanoma, and prostate cancer.^7^

Studies suggest that PDE5I utilization is widespread. A cross-sectional claims analysis noted that the proportion of men diagnosed with ED with employer-sponsored health insurance–prescribed PDE5Is increased from 18% in 2012 to 26% in 2015.^8^ Another claims data analysis found that PDE5Is constituted 75% of all prescribed ED therapies, a significantly higher frequency than all other ED therapies.^9^ PDE5Is have also become popular for enhancing male sexual performance without a medical indication,^10^ leading to increased recreational misuse. In an online epidemiologic survey of sexuality and sexual disorders in the United States, 37.5% of those with ED and 15.6% without ED cited recreational PDE5I use.^11^

Given the expected increase in the prevalence of ED and the rising numbers of men using PDE5Is, there is a need to identify and characterize safety issues related to PDE5Is used for ED and evaluate any associated safety signals in real-world settings. Postmarketing surveillance of drugs is critical for identifying safety issues. The FDA Adverse Event Reporting System (FAERS) has contributed to >50% of all postmarket safety-related label changes,^12^ highlighting the vital role in effectively monitoring and addressing emerging safety concerns. While PDE5Is are widely used for ED, previous studies of PDE5Is in FAERS were limited to specific adverse event (AE) types or conducted >10 years ago. No published studies have utilized FAERS to comprehensively evaluate the real-world safety of PDE5Is when used for ED. This study aims to provide new insights into the AEs associated with PDE5Is used for ED by investigating and comparing their characteristics and identifying any potential safety signals between 2010 and 2021.

## Methods

### Study design and database

A retrospective study was conducted on all reported AEs associated with PDE5Is in the FAERS database between January 2010 and December 2021. FAERS is a publicly available drug safety surveillance database that reports information on AEs, medication errors, and product quality complaints voluntarily submitted to the FDA by health care professionals, consumers, and manufacturers during clinical trials and postmarketing of approved drugs.^13^ An AE is defined by the FDA as

any adverse event associated with the use of a drug in humans, whether or not considered drug related, including the following: an adverse event occurring in the course of the use of a drug in professional practice; an adverse event occurring from drug overdose whether accidental or intentional; an adverse event occurring from drug abuse; an adverse event occurring from drug withdrawal; and any failure of expected pharmacological action.

AEs are classified according to the terminology of the Medical Dictionary for Regulatory Activities. AE cases are indicated as event reports that contain deidentified patient demographic information, such as age and sex, the suspected drug, indication for treatment, nature of the events, outcomes, and manufacturer information, where applicable. Since this study does not involve human participants, neither institutional review board approval nor participant consent was obtained.

The database was queried by using the generic and brand names of the PDE5Is listed in Table 1. Duplicate records based on the primary AE case report identification number (“primaryid”) and any follow-up reports were removed in accordance with the FDA’s recommendation.^14^ AEs were extracted from the database via the variable “preferred_term” or “PT.” To exclude any indication other than ED, reports were included by male sex, ED indication, age of at least 18 years old, brand name indicated for ED, strength used for ED, and oral route of administration. Reports with missing medication names were omitted from the analysis. The medication names were standardized to generic names for ease of analysis and to account for incorrect spelling. One brand name medication, Spedra, is not currently approved in the United States; however, AEs were reported in the database under the name, so they were included under avanafil.

**Table 1:** Phosphodiesterase type 5 inhibitors included in the study and their FDA approval dates.

**Generic: brand**	**Approval date**
Sildenafil: Viagra	March 27, 1998
Tadalafil: Cialis	November 21, 2003
Vardenafil	
Levitra^a^	August 19, 2003
Staxyn^b^	June 17, 2010
Avanafil	
Stendra	April 27, 2012
Spedra	Not approved in the United States

a Discontinued in United States in 2021

b Discontinued in United States on September 1, 2020

AEs were grouped by disease categories and FAERS-defined outcomes: requiring intervention to prevent permanent impairment or damage, congenital anomaly, disability, hospitalization (initial or prolonged), life-threatening, and death.^15^ AEs that were included under key disease categories are described in Table S1. The total number of AE reports, mean age, cases, AE counts, and outcomes were found by summing the counts for each drug and are expressed as numbers and percentages. The AEs are not mutually exclusive and may occur concomitantly.

### Statistical analysis

A disproportionality analysis based on proportional reporting ratio (PRR) and reporting odds ratio (ROR) was conducted to detect signals in individual PDE5Is. Two-by-two contingency tables were used to calculate the PRR and ROR with their corresponding 95% CIs for several of the most reported and clinically important events.^16^ The association between a specific drug and the target AE was determined by comparing the proportion of the AE of interest for the specific drug with that of the other PDE5Is. A positive signal was defined as a signal detected by PRR and ROR. For PRR, a signal was detected if the number of events was at least 3 and the PRR was at least 2.^17^ For ROR, a signal was detected if the lower limit of the 95% CI was >1.^18^ A chi-square test with Yates correction of at least 4 was indicative of *P* < .05. All analyses were performed with the SAS software package for Windows (version 9.4; SAS Institute Inc) or Microsoft Excel.

## Results

### Descriptive analysis

Between January 2010 and December 2021, 29 236 AEs were reported for 4 PDE5Is. Table 2 describes the characteristics of these AEs. The average age of all patients was 58.8 years. Table 3 displays the 5 most reported AEs for each PDE5I. Across all drugs, “drug ineffective” was the most reported AE (7115 reports, 24.3%). Cardiovascular (CV) events were among the 5 most reported AEs for all PDE5Is (954 reports, 3.3%). Melanoma was among the most reported AEs for sildenafil (760 reports, 4.6%) and tadalafil (637, 5.75%), while headache and migraine were among the most reported for vardenafil (27, 2.33%) and avanafil (6, 1.22%). “Erection increased” and sexual disorders were among the most reported for sildenafil and avanafil. AEs that were among the most reported for only 1 drug were back pain (347 reports, 3.13%) and fatigue (815, 7.36%) for tadalafil; dizziness (23, 1.99%) and drug administration error (39, 3.37%) for vardenafil; and flushing (6, 1.22%), visual impairment/loss (6, 1.22%), and hearing loss/impaired (6, 1.22%) for avanafil.

**Table 2:** Characteristics of adverse event reports associated with phosphodiesterase type 5 inhibitors between 2010 and 2021.^a^

**Characteristic**	**Sildenafil**	**Tadalafil**	**Vardenafil**	**Avanafil**
Adverse events	16 516 (56.49)	11 069 (37.86)	1158 (3.96)	493 (1.69)
Mean age, y	58.3	58.7	62.9	64.7
Age group				
Adolescent	1 (0.16)	2 (0.38)	0 (0)	0 (0)
Adult	415 (67.4)	361 (68.6)	148 (55.6)	55 (85.9)
Elderly	200 (32.5)	163 (31)	118 (44.4)	9 (14.1)
Outcomes				
Death	572 (9.88)	207 (5.56)	18 (4.99)	2 (2.82)
Life-threatening	36 (0.62)	34 (0.91)	7 (1.94)	0 (0)
Hospitalization	962 (16.6)	833 (22.4)	126 (34.9)	18 (25.4)
Disability	208 (3.59)	202 (5.42)	14 (3.88)	10 (14.1)
Congenital anomaly	5 (0.09)	1 (0.03)	0 (0)	0 (0)
Required intervention to prevent permanent impairment/damage	6 (0.1)	1 (0.03)	0 (0)	0 (0)
Other serious events	4002 (69.1)	2446 (65.7)	196 (54.3)	41 (57.8)

a A total of 29 236 adverse events were reported. Data are presented as No. (%) unless noted otherwise.

**Table 3:** Most reported adverse events associated with phosphodiesterase type 5 inhibitors between 2010 and 2021.^a^

**Adverse Event**	**Sildenafil**	**Tadalafil**	**Vardenafil**	**Avanafil**
Back pain		347 (3.13)		
Cardiovascular event	497 (3.01)	410 (3.7)	45 (3.89)	7 (1.42)
Dizziness			23 (1.99)	
Drug administration error			39 (3.37)	
Drug ineffective	4911 (29.73)	1396 (12.61)	424 (36.61)	334 (67.75)
Erection increased	1151 (6.97)			6 (1.22)
Fatigue		815 (7.36)		
Flushing				6 (1.22)
Headache/migraine			27 (2.33)	6 (1.22)
Hearing impairment/loss				6 (1.22)
Melanoma	760 (4.6)	637 (5.75)		
Sexual disorders	797 (4.83)			7 (1.42)
Visual impairment/blindness				6 (1.22)

a Data are presented as No. (%). Blank cells indicate *not applicable.*

There were 9947 outcome reports related to PDE5Is, with “other serious medical events” (6685 reports, 67.2%) and hospitalization (1939, 19.5%) being the most common outcomes across all drugs. Vardenafil had the highest proportion of hospitalizations (34.9%) and life-threatening outcomes (1.9%). Avanafil had the highest proportion of disability outcomes (14.1%), while sildenafil had the highest proportion of deaths (9.9%). Reports of congenital anomalies and interventions to prevent impairment were <0.1% for each drug.

### Disproportionality analysis

The disproportionality analysis detected 8 signals from the most reported and clinically important AEs among the 4 PDE5Is (Table 4). For sildenafil, there were significant signals for “drug ineffective” (PRR, 2.01 [95% CI, 1.92-2.11]; ROR, 2.44 [95% CI, 2.31-2.58]; χ^2^ = 1002.13), “erection increased” (PRR, 13.5 [10.54-17.28]; ROR, 14.44 [11.25-18.52]; χ^2^ = 752.2), sexual disorders (PRR, 3.13 [2.69-3.65]; ROR, 3.24 [2.77-3.79]; χ^2^ = 238.31), and death (PRR, 3.17 [2.5-4.01]; ROR, 3.21 [2.52-4.08]; χ^2^ = 100.45). For tadalafil, the significant signals were fatigue (PRR, 8.33 [7.05-9.84]; ROR, 8.91 [7.52-10.57]; χ^2^ = 899.67) and priapism (PRR, 3.63 [2.11-6.24]; ROR, 3.64 [2.12-6.26]; χ^2^ = 23.7). For vardenafil, the significant signal was drug administration error (PRR, 2.54 [1.84-3.52]; ROR, 2.6 [1.86-3.63]; χ^2^ = 32.07). For avanafil, the significant signal was “drug ineffective” (PRR, 3.56 [3.34-3.8]; ROR, 8.99 [7.43-10.88]; χ^2^ = 729.34).

**Table 4:** Signal strength of the most reported and clinically important adverse events associated with phosphodiesterase inhibitors.^a^

**PDE5I: adverse event**	**Cases**	**PRR (95% CI)**	**ROR (95% CI)**	**χ** ^**2**^	***P* value**
Sildenafil					
**Drug ineffective**	4911	2.01 (1.92-2.11)	2.44 (2.31-2.58)	1002.13	<.001
**Erection increased**	1151	13.50 (10.55-17.28)	14.44 (11.25-18.52)	752.2	<.001
**Sexual disorders**	797	3.13 (2.69-3.65)	3.24 (2.77-3.79)	238.31	<.001
Visual impairment/blindness	268	0.92 (0.77-1.09)	0.92 (0.77-1.09)	0.84	.2-.98
Hearing loss/impaired	149	0.64 (0.52-0.79)	0.64 (0.51-0.79)	16.38	<.001
Priapism	17	0.30 (0.17-0.52)	0.30 (0.17-0.52)	19.1	<.001
**Death**	343	3.17 (2.5-4.01)	3.21 (2.52-4.08)	100.45	<.001
Tadalafil					
Drug ineffective	1445	0.55 (0.52-0.58)	0.48 (0.45-0.51)	535.81	<.001
**Fatigue**	815	8.33 (7.05-9.84)	8.91 (7.52-10.57)	899.67	<.001
Melanoma	637	1.43 (1.29-1.59)	1.46 (1.31-1.62)	46.86	<.001
Visual impairment/blindness	210	1.22 (1.02-1.46)	1.22 (1.02-1.46)	4.68	.03-.05
Hearing loss/impaired	171	1.77 (1.43-2.19)	1.78 (1.43-2.21)	27.38	<.001
**Priapism**	42	3.63 (2.11-6.24)	3.64 (2.12-6.26)	23.7	<.001
Death	76	0.36 (0.28-0.46)	0.36 (0.28-0.46)	70.33	<.001
Vardenafil					
Drug ineffective	424	1.90 (1.76-2.06)	2.42 (2.14-2.74)	211.82	<.001
Cardiovascular events	40	1.09 (0.80-1.5)	1.10 (0.80-1.52)	0.24	.2-.98
**Drug administration error**	39	2.54 (1.84-3.52)	2.60 (1.86-3.63)	32.07	<.001
Visual impairment/blindness	13	0.66 (0.38-1.15)	0.66 (0.38-1.15)	1.88	.1-.2
Hearing loss/impaired	5	0.38 (0.16-0.91)	0.37 (0.15-0.91)	4.52	.03-.05
Priapism	0	—	—	—	—
Death	7	0.41 (0.19-0.86)	0.41 (0.19-0.86)	5.33	.03-.05
Avanafil					
**Drug ineffective**	335	3.56 (3.34-3.80)	8.99 (7.43-10.88)	729.34	<.001
Cardiovascular events	7	0.45 (0.21-0.93)	0.44 (0.21-0.92)	4.4	.03-.05
Sexual disorders	7	0.43 (0.20-0.89)	0.42 (0.20-0.89)	4.95	.03-.05
Visual impairment/blindness	6	0.72 (0.33-1.61)	0.72 (0.32-1.62)	0.31	.2-.98
Hearing loss/impaired	6	1.09 (0.49-2.43)	1.09 (0.48-2.46)	0.0001	.98-.99
Priapism	2	1.98 (0.49-8.08)	1.98 (0.48-8.14)	0.22	.2-.98
Death	1	0.14 (0.02-0.99)	0.14 (0.02-0.98)	4.55	.03-.05

Abbreviations: PDE5I, phosphodiesterase type 5 inhibitor; PRR, proportional reporting ratio; ROR, reporting odds ratio.

a The first 3 adverse events listed are the 3 most reported, and the last 4 are clinically important for each drug. The detected safety signals are in bold.

## Discussion

To the best of our knowledge, this is the first comprehensive report and disproportionality analysis on all types of AEs associated with PDE5Is used for ED in the United States. This study focused on characterizing the types of AEs and outcomes in the FAERS database and detecting signals that identify disproportionate reporting of particular AEs for 1 PDE5I as compared with the others to gain insights into potential differences among their safety profiles. The descriptive analysis found that the most reported AE for all PDE5Is was “drug ineffective” and the most reported outcome was “other serious medical events.” CV events were among the most frequent AEs across the PDE5Is. The disproportionality analysis detected 8 signals across the 4 drugs, including 4 key clinical signals: sexual disorders and death for sildenafil, priapism for tadalafil, and drug administration error for vardenafil.

The most frequent AE was “drug ineffective,” which is consistent with previous evidence showing high discontinuation rates of PDE5Is due to ineffectiveness or AEs.^19^^,^^20^ However, some patients may perceive that the drugs themselves have become less effective over time when the failure may be due to progression of underlying ED. CV events were frequently reported for all PDE5Is. CV disease (CVD) is a well-known significant risk factor for ED, with Gandaglia et al suggesting that ED and CVD are “different manifestations of the same pathophysiologic disorder.”^21^ Prior studies have described no excess risk of CVD or CV outcomes in patients treated with PDE5Is for ED.^22^ In fact, PDE5Is have been described as possibly exerting cardioprotective effects.^23^^,^^24^ Therefore, the frequency of CV event reports may be attributed to underlying CVD.

Sildenafil had 2 important signals detected in the analysis: sexual disorders and death. Sexual disorders may be signs and symptoms of comorbidities that are related to the underlying ED. The signal for death was an interesting finding, as several previous studies have indicated that PDE5Is are not associated with an increased risk of mortality. One claims analysis found that men with ED and PDE5I exposure were associated with 25% lower risk of overall mortality (hazard ratio, 0.51; *P* < .001) as compared with men with ED and no exposure to PDE5Is,^25^ while a systematic review concluded that the existing literature is insufficient to establish causality between sildenafil and mortality.^26^ However, a previous study examining CV AEs for PDE5Is in the FAERS database between 2000 and 2010 revealed that deaths represented 12.3% of the total sildenafil AEs while the current study found 9.88%.^27^ As sildenafil is the oldest and one of the most used PDE5Is, a larger proportion of patients with comorbidities that have a significantly increased risk of death may be using sildenafil (although this study’s definition of death excluded death directly related to disease). Severe outcomes such as death may be more frequent than less severe AEs. Due to the lack of studies directly examining sildenafil and mortality, an association between sildenafil and mortality cannot be ruled out. Further research is needed to investigate this potential relationship.

Tadalafil had priapism as a key clinical signal. This finding is consistent with that of Schifano et al, who found disproportionate reporting for tadalafil among drugs that are associated with priapism.^28^ However, most reports for PDE5Is were related to concomitant drugs known to cause priapism, which were taken at the same time and/or at an inappropriate intake or excessive dosage, such as trazodone, various antipsychotics, intracavernosal prostaglandin injections, and alcohol. This is consistent with the suggestion by Rezaee and Gross that drugs with a higher risk of priapism are commonly taken concomitantly with PDE5Is.^29^ Few studies have examined a direct relationship between tadalafil and priapism, so further analysis is needed to confirm these findings.

Vardenafil had a positive signal for drug administration error. The brand-name formulations of vardenafil were in orally disintegrating tablet (ODT) form, which may be the source of the drug administration errors. ODTs are commonly used for children and elderly patients who have difficulty swallowing, thereby showing their ease of use. However, an ODT can be mistaken for a film-coated tablet and may confuse some patients regarding proper administration. Additionally, patients who purchase vardenafil through illicit means may improperly take ODTs because they are unlikely to receive counseling on drug administration. Because vardenafil ODTs are primarily absorbed through local oral mucosal tissue and pregastric mucosa, men who take ODTs improperly may not experience the full effects of the drug and may report that it is ineffective. Interestingly, more regular tablet formulations of generic vardenafil are currently available on the market than ODTs, suggesting a move away from ODTs by manufacturers.^30^

Conflicting evidence exists on whether PDE5Is such as sildenafil and tadalafil increase melanoma risk. In this study, melanoma was a commonly reported AE, but no signals were detected. Two major epidemiologic studies offered different conclusions: Li et al cited an almost doubling of the melanoma hazard among sildenafil users,^31^ while Loeb et al found a modest association^32^. A meta-analysis also revealed an increased melanoma risk with PDE5I use, but the authors questioned the legitimacy of the association due to limitations such as unevaluated sun exposure.^33^ More large-scale studies, including prospective cohort studies and analyses of drug safety surveillance systems, are needed to confirm if PDE5I use is associated with melanoma. Headache and migraine are common side effects of PDE5Is, though the pathophysiologic mechanism remains unclear.^34^^,^^35^ These side effects may be related to psychiatric disorders such as depression and anxiety that often accompany ED and are associated with headaches. In this study, headache and migraine were reported frequently for only vardenafil and avanafil with no signals detected. The low reporting rates may be due to (1) PDE5I users already experiencing headaches and migraines and therefore not reporting these AEs or (2) more severe AEs being reported more frequently than less severe ones including headache and migraine.

Though rare, sudden vision loss is a serious side effect of PDE5Is, as noted in their prescribing information warnings. A FAERS analysis from 1998 to 2014 indicated that sildenafil had the most reported cases of ischemic optic neuropathy among PDE5Is, whereas avanafil had no reports.^36^ A separate FAERS analysis, spanning the initial FDA approval of PDE5Is to 2014, showed similar trends regarding retinal vascular occlusions.^37^ However, these FAERS analyses did not conduct disproportionality analyses, which would have been informative. In this study, visual impairments and blindness were among the 5 most frequent AEs for avanafil and ranked within the top 10 for all PDE5Is, with sildenafil having the greatest number of reports. While no signal was detected, caution is warranted given the potential severity of vision impairment.

While our findings contribute valuable insights into the safety of PDE5Is, it is imperative to acknowledge several limitations inherent to the FAERS data. First, FAERS relies on voluntary reporting, potentially leading to underreporting of AEs and skewing the representation of certain AEs. The quality of data may vary with potentially inaccurate, incomplete, or duplicate reports. As a result, the temporal relationship between drug exposure and AEs may not always be discernible. The absence of comprehensive patient medical histories also makes it challenging to accurately evaluate confounding factors, such as concomitant medications or underlying conditions. Though efforts were made to exclude reports with non-ED indications, some reports unrelated to ED may be present. Calculation of incidence rates is limited by the lack of a clear denominator or total drug exposures. Causality between exposure of the PDE5Is and the reported AE cannot be definitively established. Despite these limitations, the FAERS database is a valuable resource for postmarketing safety assessments.

## Conclusion

PDE5Is have been shown to significantly increase the risk of reporting certain clinically important AEs, including death for sildenafil and drug administration error for vardenafil. Because causality cannot be inferred from surveillance data, further real-world research is necessary to validate the study findings. Nonetheless, this study sheds light on previously unreported AEs and signals. Although these events do not occur often, it is imperative to continually monitor PDE5I use, from primary care to national surveillance levels, to ensure safe utilization.

## Author contributions

Y.E.S. analyzed and interpreted the data and drafted the manuscript. S.R., A.L.H., and J.J.G. contributed to the conception and design of the study. All authors critically revised the manuscript and gave final approval.

## Funding

This study was supported by Boston Scientific. The author(s) received no financial support for the research, authorship, and/or publication of this article.

## Conflicts of interest

S.R. is a full-time employee of Boston Scientific. Y.E.S. is a graduate student at the University of Cincinnati and not a Boston Scientific employee; however, she is working on a global health economics and market access project with Boston Scientific.

## Supplementary Material

pde5i_faers_supplementary_sexual_medicine_qfad059
