# Supplementary material for: Safety profile and signal detection of phosphodiesterase type 5 inhibitors for erectile dysfunction: a Food and Drug Administration Adverse Event Reporting System analysis
Source: Sex Med. 2023 Nov 29;11(5):qfad059. doi: 10.1093/sexmed/qfad059 (PMC10687329; doi:10.1093/sexmed/qfad059)
Supplement: pde5i_faers_supplementary_sexual_medicine_qfad059 [file pde5i_faers_supplementary_sexual_medicine_qfad059.docx]

**Supplementary Table 1. Descriptions of key disease-specific adverse event categories**

| **Adverse event types** | **Preferred terms** |
| --- | --- |
| Back pain | Back pain  Flank pain |
| Cardiovascular events | Acute coronary syndrome  Aneurysm  Angina pectoris  Angina unstable  Angiopathy  Angioplasty  Aortic aneurysm  Aortic dissection  Aortic stenosis  Aortic thrombosis  Aortic valve calcification  Aortic valve disease  Aortic valve incompetence  Arrhythmia  Arterial disorder  Arterial insufficiency  Arterial occlusive disease  Arterial rupture  Arterial spasm  Arterial stenosis  Arterial thrombosis  Arteriosclerosis  Arteriosclerosis coronary artery  Arteriospasm coronary  Arteriovenous malformation  Artery dissection  Atrial fibrillation  Atrial flutter  Atrial septal defect repair  Atrioventricular block complete  Atrioventricular block first degree  Atrioventricular block second degree  Bradycardia  Bundle branch block left  Bundle branch block right  Cardiac ablation  Cardiac arrest  Cardiac death  Cardiac discomfort  Cardiac disorder  Cardiac failure  Cardiac failure acute  Cardiac failure chronic  Cardiac failure congestive  Cardiac fibrillation  Cardiac flutter  Cardiac operation  Cardiac pacemaker insertion  Cardiac stress test abnorm  Cardiac valve disease  Cardio-respiratory arrest  Cardiogenic shock  Cardiomegaly  Cardiomyopathy  Cardiopulmonary failure  Cardiovascular disorder  Cardioversion  Carotid arteriosclerosis  Carotid artery aneurysm  Carotid artery dissection  Carotid artery occlusion  Carotid artery stenosis  Carotid artery thrombosis  Circulatory collapse  Coagulopathy  Coronary artery disease  Coronary artery occlusion  Coronary artery stenosis  Deep vein thrombosis  Disseminated intravascular coagulation  Electrocardiogram abnormal  Electrocardiogram ST segment  Embolism  Hypertension  Hypertensive crisis  Hypotension  Infarction  Intermittent claudication  Intracardiac thrombus  Ischaemia  Left ventricular hypertrophy  Mitral valve disease  Myocardial infarction  Orthostatic hypotension  Palpitations  Pericardial effusion  Peripheral ischaemia  Peripheral vascular disorder  Sudden cardiac death  Supraventricular tachycardia  Tachyarrhythmia  Tachycardia  Tachycardia foetal  Thrombophlebitis superficial  Thrombosis  Vascular calcification  Vascular occlusion  Vascular stent stenosis  Vasculitis  Vein rupture  Venous thrombosis  Ventricular arrhythmia  Ventricular tachycardia |
| Death | Accidental death  Apparent death  Death  Sudden death |
| Drug administration error | Contraindicated product administered  Counterfeit drug administered  Counterfeit product administered  Drug administered at inappropriate site  Drug administered to patient of inapprop  Drug dose omission  Drug titration error  Expired drug administered  Extra dose administered  Inappropriate schedule of drug administr  Inappropriate schedule of product admini  Incorrect dosage  Incorrect dosage administered  Incorrect dose administered  Incorrect drug administration dura  Incorrect product admini  Incorrect route of administration  Incorrect route of drug administrat  Intentional dose omission  Poor quality drug administ  Product administered to patient of inapp  Wrong technique in drug usage process |
| Erection increased | Painful erection  Spontaneous penile erection |
| Fatigue | Asthenia  Chronic fatigue syndrome  Fatigue |
| Flushing | Flushing  Hot flush |
| Headache/migraine | Basilar migraine  Cluster headache  Migraine  Migraine with aura  Primary headache associated with s  Sinus headache  Thunderclap headache |
| Hearing impaired/loss | Deafness  Deafness bilateral  Deafness neurosensory  Deafness transitory  Deafness unilateral  Hearing disability  Hearing impaired  Sudden hearing loss |
| Melanoma | Acral lentiginous melanoma stage III  Choroid melanoma  Desmoplastic melanoma  Gastrointestinal melanoma  Iris melanoma  Lentigo maligna melanoma  Malignant melanoma in situ  Malignant melanoma stage I  Malignant melanoma stage II  Malignant melanoma stage III  Malignant melanoma stage IV  Melanoma recurrent  Metastatic malignant melanoma  Nodular melanoma  Superficial spreading melanoma |
| Sexual disorders | Anorgasmia  Ejaculation disorder  Premature ejaculation  Ejaculation failure  Ejaculation delayed  Ejaculation disorder  Ejaculation failure  Spontaneous ejaculation  Painful ejaculation  Ejaculation delay  Retrograde ejaculation  Aspermia  Disturbance in sexual arousal  Dyspareunia  Excessive masturbation  Fertility increased  Gynaecomastia  Hypersexuality  Infertility  Infertility male  Libido decreased  Libido disorder  Libido increased  Loss of libido  Male orgasmic disorder  Orgasm abnormal  Orgasmic sensation decreased  Compulsive sexual behaviour  Sexual activity increased |
| Visual impairment/blindness | Altered visual depth perception  Astigmatism  Blindness  Blindness transient  Blindness unilateral  Cataract  Cataract nuclear  Cataract operation  Colour blindness acquired  Colour vision tests abnormal  Dyschromatopsia  Erythropsia  Hallucination  Hallucination, visual  Halo vision  Loss of visual contrast sensitivity  Night blindness  Sudden visual loss  Vision blurred  Visual acuity red  Visual acuity reduced  Visual brightness  Visual field defect  Visual field tests abnormal |
